# Enhanced Go and NoGo Learning in Individuals With Obesity

**DOI:** 10.3389/fnbeh.2020.00015

**Published:** 2020-02-14

**Authors:** Jana Kube, Kathleen Wiencke, Sandra Hahn, Arno Villringer, Jane Neumann

**Affiliations:** ^1^Department of Neurology, Max Planck Institute for Human Cognitive and Brain Sciences, Leipzig, Germany; ^2^Leipzig University Medical Center, IFB Adiposity Diseases, Leipzig, Germany; ^3^Faculty 5–Business, Law and Social Sciences, Brandenburg University of Technology Cottbus–Senftenberg, Cottbus, Germany; ^4^Clinic of Cognitive Neurology, University Hospital Leipzig, Leipzig, Germany; ^5^Berlin School of Mind and Brain, Mind and Brain Institute, Humboldt-University, Berlin, Germany; ^6^Department of Medical Engineering and Biotechnology, University of Applied Sciences, Jena, Germany

**Keywords:** obesity, prediction error, reinforcement learning, instrumental, Pavlovian

## Abstract

Overeating in individuals with obesity is hypothesized to be partly caused by automatic action tendencies to food cues that have the potential to override goal-directed dietary restriction. Individuals with obesity are often characterized by alterations in the processing of such rewarding food, but also of non-food stimuli, and previous research has suggested a stronger impact on the execution of goal-directed actions in obesity. Here, we investigated whether Pavlovian cues can also corrupt the learning of new approach or withdrawal behavior in individuals with obesity. We employed a probabilistic Pavlovian-instrumental learning paradigm in which participants (29 normal-weight and 29 obese) learned to actively respond (Go learning) or withhold a response (NoGo learning) in order to gain monetary rewards or avoid losses. Participants were better at learning active approach responses (Go) in the light of anticipated rewards and at learning to withhold a response (NoGo) in the light of imminent punishments. Importantly, there was no evidence for a stronger corruption of instrumental learning in individuals with obesity. Instead, they showed better learning across conditions than normal-weight participants. Using a computational reinforcement learning model, we additionally found an increased learning rate in individuals with obesity. Previous studies have mostly reported a lower reinforcement learning performance in individuals with obesity. Our results contradict this and suggest that their performance is not universally impaired: Instead, while previous studies found reduced stimulus-value learning, individuals with obesity may show better action-value learning. Our findings highlight the need for a broader investigation of behavioral adaptation in obesity across different task designs and types of reinforcement learning.

## Introduction

Over-consumption of high-caloric food is thought to be one of the main contributing factors to the development of human obesity. Affected individuals often maintain their dysfunctional eating behavior over long periods of time, even in the light of short- and long-term negative consequences. Thus, overeating shows a paradoxical effect: despite the negative consequences of unhealthy nutrition and the motivation to change their eating behavior, individuals with obesity often struggle with effective behavioral change (Andreyeva et al., 2010).

It has been hypothesized that this may in part be caused by automatic action tendencies to rewarding food cues that increase the likelihood of consumption (Johnson, 2013; Rangel, 2013). Pavlovian cues signaling the prospect of a (food) reward typically induce feelings of desire (wanting) and active approach behavior to obtain the reward. These hard-wired responses occur even when this behavior may not be beneficial in the current situation, i.e., they can corrupt goal-directed actions. Consequently, it may be easier to learn and execute an active approach response in the prospect of a reward, while the threat of punishment may foster action inhibition (Guitart-Masip et al., 2011, 2012; Huys et al., 2011; Cavanagh et al., 2013; Lindström et al., 2015). Evidence suggests that this corruption of goal-directed behavior could be amplified in some individuals. For instance, Garofalo and di Pellegrino (2015) found that individuals with a strong focus on reward-predicting cues show a stronger bias of instrumental choice behavior by these stimuli. Similarly, individuals who rate rewards as more valuable show a stronger distortion of goal-directed behavior than individuals who rate them as less valuable (Lehner et al., 2017b).

In obesity research, several theories argue that obesity is characterized by an increased responsivity to rewarding food cues (Berridge et al., 2010; Chen et al., 2018; Stice and Burger, 2018). In support of this, multiple studies have consistently found increased neural activation to palatable food cues (Stice et al., 2008; García-García et al., 2014; Feldstein Ewing et al., 2017) as well as a potentially stronger reinforcing efficacy (Saelens and Epstein, 1996). This increased responsiveness to cues of reward may similarly affect the execution of goal-directed behavior. Indeed, Horstmann et al. (2015b) reported that food reward cues were able to trigger approach behavior in individuals with obesity even after they had consumed the food *ad libitum* and reported being less motivated to obtain it. Together, these results suggest that individuals with obesity may show a stronger corruption of goal-directed behavior by “stimulus-driven” automatic action tendencies.

In dynamic environments not only the execution, but also the learning of beneficial actions may be corrupted by the presentation of salient Pavlovian cues. To date, most studies in individuals with obesity have focused on tasks that require an active choice of more advantageous (reward or punishment avoidance predicting) choice options. They have consistently found individuals with obesity to be impaired in this type of reinforcement learning (Coppin et al., 2014; Kastner et al., 2017; Mathar et al., 2017; Kube et al., 2018). However, by focusing on active choice responses these tasks have largely ignored the inherent coupling of reward—approach and punishment—inhibition tendencies. Learning and integrating an inhibitory response in the light of a prospective reward may be more challenging to individuals with obesity, who show an increased responsiveness to cues of reward (Berridge et al., 2010; Chen et al., 2018; Stice and Burger, 2018).

Importantly, early research focused on the processing of palatable food cues. However, the same mechanisms likely also affect behavior outside of the food context. Ample evidence suggests that food and non-food rewards are processed in largely overlapping neural areas (Levy and Glimcher, 2012; Bartra et al., 2013; Clithero and Rangel, 2013; Sescousse et al., 2013) and individuals with obesity also show altered neural responses to non-food reinforcers (Balodis et al., 2013; Opel et al., 2015).

Here, we investigate behavioral differences between normal-weight and obese individuals in instrumental learning and determine the influence of Pavlovian cues on learning performance in individuals with obesity. A probabilistic Pavlovian-instrumental learning paradigm adopted from Guitart-Masip et al. (2012) was employed. Participants were asked to learn correct approach (Go) or inhibitory (NoGo) responses to cues that predicted reward or punishment. Monetary reinforcement stimuli (gains and losses) were used as they show obesity-related brain activity alterations that are similar to those found for food-stimuli in other studies (e.g., Balodis et al., 2013; García-García et al., 2014; Opel et al., 2015), and may be less prone to momentary evaluation fluctuations than food cues (Field et al., 2016). In addition to the primary task, two brief control experiments were carried out. These tasks separately evaluated whether individuals with obesity have more general alterations in basic Pavlovian- or instrumental learning processes. Behavioral measurements were complemented by computational modeling in order to differentiate Pavlovian and instrumental influences on learning performance (Guitart-Masip et al., 2012).

We hypothesized that individuals with obesity will show a stronger influence of reward-predicting Pavlovian cues, on the learning of goal-directed actions, than normal-weight participants. Specifically, we expected enhanced learning performance when learning an active approach response (reward Go) and impaired performance when learning to withhold an active response while anticipating a monetary gain (reward NoGo). With the two additional monetary loss conditions (punishment Go and punishment NoGo) we explored the impact of punishment cues on instrumental learning.

## Materials and Methods

### Participants

Sixty-three participants were recruited from the database of the Max Planck Institute for Human Cognitive and Brain Sciences in Leipzig, Germany. All participants underwent an initial telephone screening to evaluate inclusion and exclusion criteria. Inclusion criteria encompassed age between 18 and 35 years, as well as BMI between 18.5 and 24.9 kg/m^2^ for normal-weight participants and equal to or above 30.0 kg/m^2^ for individuals with obesity. Participants were not selected to participate in the study if they reported currently smoking, the use of psychoactive medication or illegal drugs, excessive alcohol consumption, a history of neuropsychiatric disease, diabetes, or thyroid disease. Upon participation, we excluded two participants (one normal-weight male, one obese female) who reported to be normal-weight/obese at the time of recruitment, but fell outside of our predefined BMI criteria at the time of measurement. Further, two participants (one obese female, one normal-weight female) were excluded due to lack of task compliance and one participant (normal-weight male) was excluded because of current depressive symptomatology. The final sample thus consisted of 29 normal-weight participants (14 female) and 29 participants with obesity (14 female).

Prior to the main experiment all participants completed a digit span working memory task from the Wechsler Memory Scale—Revised (WMS-R, Wechsler, 1987). This was done as previous studies have suggested an influence of working memory on reinforcement learning (Collins and Frank, 2012) and cue conditioning (Coppin et al., 2014). Additionally, all participants completed a battery of questionnaires to assess personality, clinical characteristics as well as eating behavior, which may contribute to differences in learning performance. Specifically, this encompassed Beck's Depression Inventory (BDI, Beck and Steer, 1987), the Barrat Impulsiveness Scale—Short Form (BIS-15, Spinella, 2007), the BIS/BAS Scales (Carver and White, 1994), and the Three Factor Eating Questionnaire (TFEQ, Stunkard and Messick, 1985). Weight, height, and waist-circumference were obtained subsequent to the main experiments in accordance with the measurement recommendations of the WHO Expert Consultation (2008).

All participants gave written informed consent prior to their participation and received a fixed reimbursement of 9€/h with an average study duration of 2 h. Additionally, all participants received a monetary bonus depending on their performance in the Pavlovian-instrumental learning task (10% of their net outcome; on average 2.28€). The study was carried out in accordance with the Declaration of Helsinki and was approved by the ethics committee of the University of Leipzig.

### Learning Tasks

**Figure 1:**
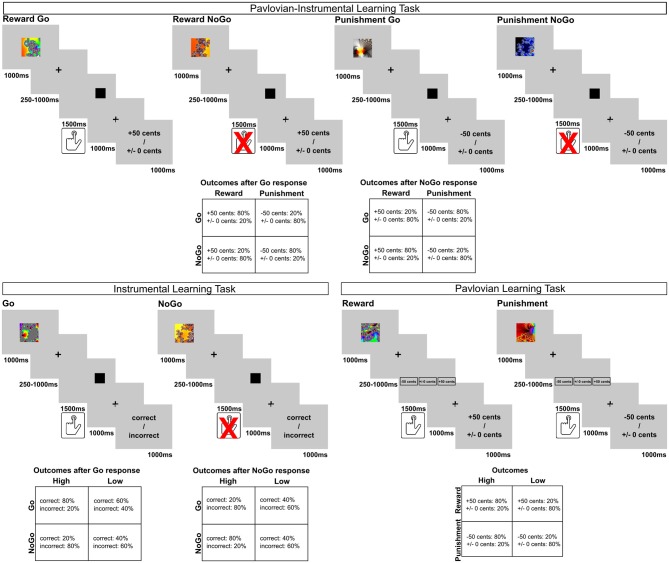
Trial structure and outcome contingencies of the experiments.

Three independent learning tasks were employed in the current study. Initially, all participants performed a Pavlovian-instrumental learning task. Subsequently, they completed two additional control tasks in a pseudorandomized order to separately evaluate basic group differences in instrumental and Pavlovian learning. All tasks were presented using Presentation® software (Version 16.5, Neurobehavioral Systems, Inc., Berkeley, CA, www.neurobs.com). Trial structure and timing of all tasks are displayed in Figure 1.

#### Main Task: Pavlovian-Instrumental Learning (PIL)

To examine obesity-related alterations in Pavlovian influences on instrumental learning, we used a task developed by Guitart-Masip et al. (2012). In this task each trial is comprised of three main events: the presentation of a cue, target detection, and the presentation of a financial outcome. At the beginning of a trial one of four fractal cues was presented. The cues were randomly assigned to the four different trial types. They indicated whether the participants were subsequently expected to respond to a target stimulus (Go) or not (NoGo), and which financial outcome was at stake in the current trial (reward or punishment). Following the cue and a variable delay period, a target stimulus (black square) appeared on the screen. The participants could then either perform the target detection task (Go response) or wait until the target stimulus disappeared (NoGo response). If they decided to act, their task was to press a button to indicate where the target was presented on the screen. They were instructed to learn the correct response associated with each cue from the trial outcomes, which were presented after a second delay period.

In sum, the task orthogonalizes the influences of action (Go, NoGo responses) and outcome valence (reward, punishment) by including four trial types: reward Go, reward NoGo, punishment Go, and punishment NoGo. In Go-Trials participants learned to respond to the target in order to achieve a beneficial trial outcome, while in NoGo-Trials the correct response was to refrain from a button press. This instrumental learning process was manipulated by the addition of two potential outcome valences. In reward trials, correct (Go or NoGo) responses were rewarded by a monetary gain of 50 cents. In punishment trials, correct (Go or NoGo) responses avoided a monetary loss of 50 cents.

The outcome-contingencies were probabilistic. Correct responses in reward trials lead to a monetary gain in 80% of the trials and a financially neutral feedback (±0 cents) in 20% of the trials. Conversely, 80% of incorrect responses were followed by neutral feedback, while 20% were followed by a monetary gain. Similarly, in the punishment condition, correct responses were associated with an 80% probability of avoiding a monetary loss and only a 20% risk of losing 50 cents. Incorrect responses were associated with the reversed contingencies. The participants were informed about the probabilistic nature of the task.

The task included 240 trials (60 trials per trial type) and had an average duration of ~26 min. Trial order was randomized in blocks of 80 trials to ensure a roughly equal number of trials per trial type at each stage of the experiment. Participants performed a practice block consisting of 16 trials to familiarize them with the trial structure, response mode, and probabilistic outcome presentation. They were informed that at the end of the experiment they would receive a monetary bonus depending on their net outcome in the task, but did not know the exact proportion of the net outcome they would gain.

#### Control Task 1: Instrumental Learning (INST)

In addition to the primary task, we developed a simple instrumental learning task to evaluate basic group differences in instrumental learning, without the influence of an anticipated monetary gain or loss. Similar to the *PIL*, a fractal cue was presented at the beginning of each trial, followed by a delay period, and a subsequent target stimulus. Participants learned to respond to the target by pressing a button (Go trials) or withholding their response (NoGo trials). However, here the outcome was not differentiated in terms of valence (reward vs. no reward, punishment vs. avoidance of punishment). This was done to minimize the motivating effect of monetary reinforcement on the instrumental learning process. Instead, correct responses were usually followed by written feedback on the screen saying “correct,” while incorrect responses were usually followed by feedback saying “incorrect.”

Similar to the other tasks, feedback was given probabilistically. To increase task complexity, we employed two different probability conditions: In high probability trials, 80% of correct (Go, NoGo) responses were followed by “correct” feedback, while 20% of the correct responses were followed by false “incorrect” feedback. Consequently, 80% of incorrect responses were followed by “incorrect feedback” and 20% of incorrect responses were followed by false “correct” feedback. In low probability trials only 60% of correct responses were followed by “correct” feedback, while 40% of correct responses were followed by false “incorrect” feedback and *vice versa*.

The task thus included 4 trial types (Go high, Go low, NoGo high, NoGo low), which were signaled by four different fractal cues. Participants were again instructed to learn the correct response associated with each cue using the feedback. They were informed about the probabilistic nature of the task. Participants performed 80 trials (20 trials per type) that were randomized in blocks of 40 trials to ensure a roughly equal number of trials per trial type at each stage of the experiment. The average duration of the task was ~9 min. It was preceded by a practice block of 12 trials.

#### Control Task 2: Pavlovian Learning (PAVLO)

Additionally, we investigated basic group differences in probabilistic stimulus-value learning between normal-weight and obese participants. In this brief task, participants were again presented with one of four different fractal cues at the beginning of each trial. Following a variable delay, they were then asked to predict which outcome would follow at the end of the trial (options: −50 cents, ±0 cents, +50 cents). Subsequently, the trial outcome was presented. It was stressed to the participants that the trial outcome did not depend on their responses, but on the cue presented at the beginning of the trial. Thus, participants had to observe the cue-outcome contingencies over the course of the task in order to make correct predictions.

Similar to *PIL*, the task included two outcome valences: either a monetary reward of 50 cents, or a monetary loss of 50 cents. Feedback was delivered probabilistically and two probability conditions were employed. In high probability trials the fractal cue was followed by a reward (reward high) or punishment (punishment high) in 80% of the trials and in only 20% of the trials it was followed by financially neutral feedback. In “low probability” trials these contingencies were reversed, such that a reward (or punishment) was presented in only 20% of the trials and feedback was neutral otherwise. The task thus consisted of 4 trial types: reward high, reward low, punishment high, punishment low with 20 trials per block. The trials were randomized in blocks of 40 trials to ensure a roughly equal number of trials per condition at each stage of the experiment. The average task duration was ~9 min. Participants again performed a practice block of 12 trials and were instructed about the probabilistic nature of the task.

### Statistical Analyses

The statistical analyses were carried out using IBM SPSS Statistics 24 (Armonk, NY, USA) with a level of significance at *p* < 0.05.

For the analyses of learning performance in the three tasks, we applied a generalized estimating equations approach (GEE). GEE is an extension of the generalized linear model that accounts for the dependency of observations by specifying a working correlation structure and is suitable for linear, ordinal, and categorical outcome variables (Zeger and Liang, 1986). We computed GEE models for count data with a Poisson distribution, log link function, and unstructured or exchangeable working correlation matrix. For all analyses we first set up a full factorial model employing all possible main and interaction effects. Subsequently, we trimmed down the model by progressively dropping model effects that did not yield a significant influence on our outcome variable (Crawley, 2007). To do that, we inspected the parameter estimates of each model and removed the least significant terms, starting with non-significant interaction terms. We then used the corrected quasi likelihood under independence model criterion (QICC) to compare the model fit of the current and the reduced model and only retained the reduced model if it provided a better model fit. This was repeated until a final model was found, which provided the best model fit with the fewest number of predictors as determined with QICC goodness of fit statistics. Bonferroni-corrected *t*-tests were utilized as *post-hoc* tests where the GEE indicated a significant main or interaction effect. Cohen's d was calculated as a measure of effect size for all pairwise comparisons.

### Reinforcement Learning Model

Guitart-Masip et al. (2012, 2014) describe a set of reinforcement learning models that, depending on the incorporated parameters, model the putatively different instrumental and Pavlovian influence on task performance. Through model comparison, the authors identified the model that best fit their behavioral data of healthy individuals. Given that our data was obtained from an identical task, we applied the best fitting and most parsimonious model identified by Guitart-Masip et al. (2014).

The model assigns to each action *a*_*t*_ on trial *t* a choice probability *p*(*a*_*t*_|*s*_*t*_) based on action propensities *W*_*t*_(*a*_*t*_, *s*_*t*_) with stimulus *s*_*t*_ presented on that trial:

$$\begin{array}{l} p\left(a_{t}|s_{t}\right)=\left[\frac{exp\left(W_{t}\left(a_{t},s_{t}\right)\right)}{\sum_{a}exp\left(W_{t}\left(a,s_{t}\right)\right)}\right]\left(1-\xi \right)+\frac{\xi }{2} \end{array}$$

The model includes an irreducible noise parameter ξ, which implements a choice probability close to chance when approaching 1. This represents a scenario where an individual's choice is largely independent of the presented stimulus and hence highly inconsistent.

Action propensities were constructed as follows:

$$W_{t}\left(a_{t},s_{t}\right)=\{\begin{array}{ll} Q_{t}\left(a_{t},s_{t}\right)+b+\pi V_{t}\left(s_{t}\right) & if\;a_{t}=go\\ \;Q_{t}\left(a_{t},s_{t}\right) & else. \end{array}\;$$

Here, *Q*_*t*_(*a*_*t*_, *s*_*t*_) implements the instrumental component depending on action-stimulus pairs in the current trial (*a*_*t*_, *s*_*t*_) and is updated by a Rescorla-Wagner-like update equation

$$\begin{array}{l} Q_{t}\left(a_{t},s_{t}\right)=Q_{t-1}\left(a_{t},s_{t}\right)+\varepsilon \left(\rho r_{t}-Q_{t-1}\left(a_{t},s_{t}\right)\right) \end{array}$$

with learning rate ε, effectiveness parameter ρ, and reinforcement *r* ∈ {1, 0, −1} for reward, neutral feedback, and punishment, respectively. Parameter ρ is divided into ρ_*R*_ and ρ_*P*_ for reward and punishment trials, respectively. Thus, the model treats the effective size of reinforcement differentially for the two outcome categories.

The parameter *b* implements a constant action bias, accounting for individuals' tendency to perform the target detection task independent of the presented stimulus. *V*_*t*_(*s*_*t*_) represents the state value of stimulus *s*_*t*_ and is updated in a similar manner as the instrumental component. It is scaled by the Pavlovian parameter π which determines the strength of Pavlovian influences on the action propensity:

$$\begin{array}{l} V_{t}\left(s_{t}\right)=V_{t-1}\left(s_{t}\right)+\varepsilon \left(\rho r_{t}-V_{t-1}\left(s_{t}\right)\right). \end{array}$$

Thus, in total the model comprises 6 free parameters to be estimated for each subject.

Following Huys et al. (2011) and Guitart-Masip et al. (2012, 2014) model parameters were determined based on behavioral data independently for each participant, by calculating maximum posterior (MAP) estimates. The procedure uses maximum likelihood estimation (MLE) on the population level for prior distributions over unbounded model parameters. This means, we used Gaussian priors for the action bias, Pavlovian parameter, and the effectiveness parameters as well as Beta priors for learning rate and the irreducible noise parameter, all with mean and variance equal to the mean and variance obtained from MLEs.

On each iteration the posterior distribution over the whole sample for each parameter was used to specify the prior of the individual parameters on the next iteration. Differences between normal-weight individuals and individuals with obesity were assessed after model fitting by two sample *t*-tests for the two sets of MAPs.

## Results

### Participant Characteristics

Demographic and anthropometric characteristics, working memory performance as well as personality measures are presented in Table 1. Expectedly, individuals with obesity had a significantly higher BMI [*t*_(56)_ = 12.010, *p* < 0.001] and waist-circumference [*t*_(56)_ = 10.320, *p* < 0.001] than normal-weight participants. The groups did not significantly differ in age [*t*_(56)_ = −0.272, *p* = 0.787], sex distribution (X^2^ = 0.000, *p* = 1), school education levels (X^2^ = 0.000, *p* = 1), or working memory scores [*t*_(56)_ = −0.229, *p* = 0.820]. However, they differed in higher education levels (X^2^ = 9.544, *p* = 0.049). More obese participants were currently enrolled in a higher education program, while more normal-weight participants had already completed a Bachelor's degree. Additionally, individuals with obesity exhibited lower behavioral inhibition scores [BIS; *t*_(56)_ = −2.177, *p* = 0.034] and higher disinhibited eating [TFEQ-Dis; *t*_(56)_ = 3.750, *p* < 0.001] than normal-weight participants.

**Table 1:** Sample characteristics.

	**Participants with** **obesity** ***n* = 29**	**Participants without** **obesity** ***n* = 29**	**Test statistic**
**Demographics**			
Female/male	14/15	14/15	*X*^2^ = 0.000, *p* = 1
Age	26.79 ± 3.70	27.03 ± 3.04	*t*_(56)_ = −0.272, *p* = 0.787
A levels	28	28	*X*^2^ = 0.000, *p* = 1
Higher education	5/10/5/2/7	2/3/5/8/11	*X*^2^ = 9.544, ***p*** **=** **0.049**
**Anthropometrics**			
BMI	36.98 ± 6.24	22.60 ± 1.60	*t*_(56)_ = 12.010, ***p*** **<** **0.001**
WC	103.07 ± 13.71	74.69 ± 5.61	*t*_(56)_ = 10.320, ***p*** **<** **0.001**
**Tests and questionnaires**			
DS Forward	10.34 ± 2.06	10.52 ± 1.86	*t*_(56)_ = −0.334, *p* = 0.739
DS Backward	8.28 ± 1.65	8.31 ± 2.16	*t*_(56)_ = −0.068, *p* = 0.946
DS Total	18.62 ± 3.26	18.83 ± 3.62	*t*_(56)_ = −0.229, *p* = 0.820
BDI	5.14 ± 4.24	4.00 ± 3.58	*t*_(56)_ = 1.138, *p* = 0.260
BIS/BAS-BIS	2.75 ± 0.58	3.04 ± 0.41	*t*_(56)_ = −2.177, ***p*** **=** **0.034**
BIS/BAS-BAS	3.11 ± 0.38	3.18 ± 0.30	*t*_(56)_ = −0.742, *p* = 0.461
BIS-15	32.34 ± 6.32	30.21 ± 6.50	*t*_(56)_ = 1.269, *p* = 0.210
TFEQ Dis	8.17 ± 3.56	5.14 ± 2.52	*t*_(56)_ = 3.750, ***p*** **<** **0.001**
TFEQ Restraint	6.76 ± 5.02	5.38 ± 4.10	*t*(_56)_ = 1.146, *p* = 0.257
TFEQ Hunger	5.66 ± 4.06	5.14 ± 3.30	*t*_(56)_ = 0.533, *p* = 0.596

*A levels, number of participants with A levels education; Higher education, number of participants with no higher education/ongoing higher education/vocational training/Bachelor's degree/Master's degree; BMI, Body Mass Index in kg/m^2^; WC, Waist-circumference in cm; DS Forward, Wechsler Memory Scale–Revised, Subtest Digit Span Forward; DS Backward, Wechsler Memory Scale—Revised, Subtest Digit Span Backward; DS Total, Wechsler Memory Scale—Revised, Digit Span Forward + Digit Span Backward; BDI, Beck‘s Depression Inventory; BIS/BAS-BIS, Behavioral Inhibition/Behavioral Activation Scale—Subscale Behavioral Inhibition; BIS/BAS-BAS, Behavioral Inhibition/Behavioral Activation Scale—Subscale Behavioral Activation; BIS-15, Barrat Impulsiveness Scale—Short Form; TFEQ Dis, Three Factor Eating Questionnaire—Subscale Disinhibition; TFEQ Restraint, Three Factor Eating Questionnaire—Subscale Cognitive Restraint; TFEQ Hunger, Three Factor Eating Questionnaire—Subscale Hunger. Values represent mean ± SD. Independent samples t-tests are reported for continuous variables, while X^2^ is reported for comparisons of categorical data. Results significant at p < 0.05 (two-tailed) are printed in bold letters*.

### PIL

**Figure 2:**
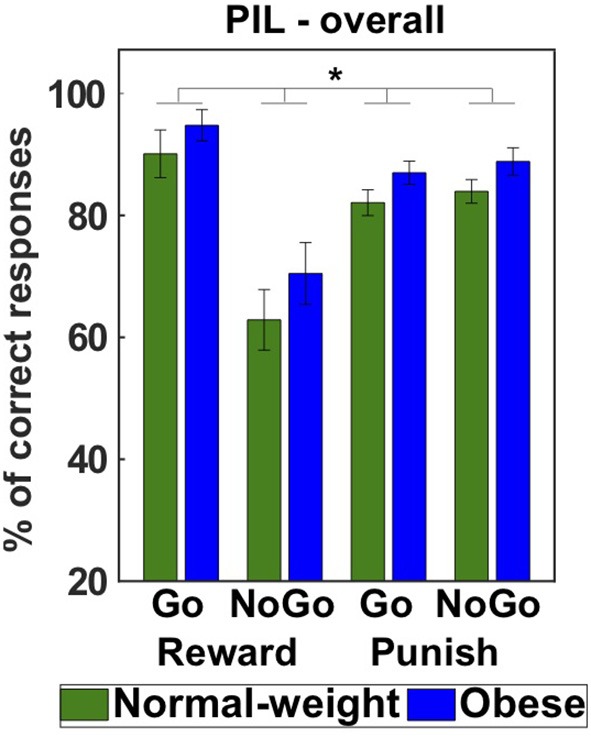
Behavioral results of the Pavlovian-instrumental learning task. The mean percentage of correct responses across Reward Go, Reward NoGo, Punish Go, and Punish NoGo trials shows that individuals with obesity achieved a higher learning performance across conditions than normal-weight participants (main effect of group) *p < 0.05.

To investigate the influence of anticipated reward and punishment on instrumental learning performance we analyzed the number of correct responses in each learning condition in the *PIL* task. We set up a GEE model including all main and interaction effects (full model with QICC = 999.026) involving the predictors valence (reward, punishment), action (Go, NoGo), group (normal-weight, obese), and sex (male, female) and subsequently reduced the model (see section Materials and Methods for a more detailed description). The final reduced model (QICC = 980.465) included significant main effects of action [Wald X^2^ = 20.780, *p* < 0.001], valence (Wald X^2^ = 7.545, *p* = 0.006) as well as a significant action by valence interaction (Wald X^2^ = 25.759, *p* < 0.001). In line with previous studies, we found a significantly higher learning performance during reward Go trials than punishment Go trials (*p* = 0.001, *d* = 0.47) and a better performance for punishment NoGo than reward NoGo learning trials (*p* < 0.001, *d* = 0.64). Further, learning to approach a reward in reward Go trials was associated with a significantly higher learning performance than learning to withhold a response in reward NoGo trials (*p* < 0.001, *d* = 0.72). There was, however, no evidence for a modulation of this effect by group (3-way interaction of Group × Valence × Action: Wald X^2^ = 0.206, *p* = 0.650), suggesting similar influences of Pavlovian cues on instrumental learning performance in normal-weight and obese participants. Rather, we found a significant main effect of group (Wald X^2^ = 3.924, *p* = 0.048, *d* = 0.50, Figure 2). Surprisingly this suggests a higher learning performance in individuals with obesity than normal-weight participants across conditions. Learning performance was not significantly modulated by sex (Wald X^2^ = 0.000, *p* = 0.987) nor a combined influence of sex and obesity weight status (2-way interaction of Sex × Group: Wald X^2^ = 0.007, *p* = 0.935).

Next, we set up additional models to test whether differences in learning performance were affected by personality characteristics and alterations in working memory. We found no evidence for a significant mediation, suggesting that obesity-related alterations in learning performance were not related to differences in personality or working memory capacity. For a more detailed description see the Supplementary Material.

### INST

**Figure 3:**
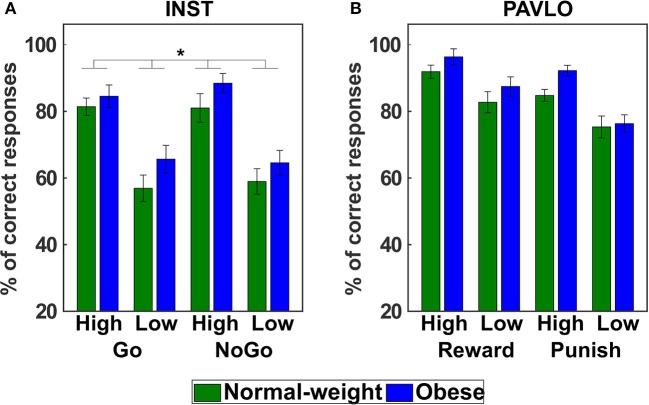
Behavioral results of the instrumental and Pavlovian control tasks. **(A)** The mean percentage of correct responses in the instrumental learning task shows that individuals with obesity achieved a higher learning performance across conditions than normal-weight participants (main effect of group). **(B)** In the Pavlovian learning task, there was no evidence for a differential learning performance in normal-weight and obese individuals. Error bars represent standard errors of the mean taking into account the within-subject design (Cousineau, 2005; Morey, 2008). *p < 0.05 (two-tailed).

In the next step, we analyzed the number of correct responses in the instrumental learning paradigm utilizing a GEE model with all main and interaction effects (full model with QICC = 424.420) involving the predictors action (Go, NoGo), probability (high, low), group (normal-weight, obese), and sex (male, female). After model reduction, the final model (QICC = 416.422) included significant main effects of probability (Wald X^2^ = 36.616, *p* < 0.001) and group (Wald X^2^ = 4.492, *p* = 0.034, Figure 3A). Learning performance was significantly better for trials that had a high compared to a low probability of providing correct feedback (*d* = 0.92). Interestingly, individuals with obesity again showed a significantly higher learning performance than normal-weight participants across conditions (*d* = 0.47).

### PAVLO

For the analysis of stimulus-outcome learning we examined the number of correct predictions, i.e., the number of trials in which a participant predicted the most frequently presented outcome for a respective stimulus. This information was subjected to a GEE model (full model with QICC = 217.216) with the predictors valence (reward, punishment), probability (high, low), group (normal-weight, obese), and sex (male, female). The final reduced model (QICC = 205.293) showed significant main effects of valence (Wald X^2^ = 23.399, *p* < 0.001) and probability (Wald X^2^ = 36.497, *p* < 0.001) as well as significant interactions of sex and valence (Wald X^2^ = 7.127, *p* = 0.008) and sex and probability (Wald X^2^ = 4.284, *p* = 0.038). Bonferroni-corrected *post-hoc* tests did not yield any significant differences. At an uncorrected level, male participants were better than female participants in the punishment condition (*p*_uncorr_ = 0.021, *p*_corr_ = 0.168, *d* = 0.62) and in the low reinforcement probability trials (*p*_uncorr_ = 0.041, *p*_corr_ = 0.331, *d* = 0.55). As opposed to the other tasks, there was no significant difference between normal-weight and obese participants (main effect of Group: Wald X^2^ = 1.572, *p* = 0.210, Figure 3B).

### Reinforcement Learning Models

From the computational model adopted from Guitart-Masip et al. (2014), MAP estimates for the 6 parameters of interest were obtained for each participant (Table 2). Bonferroni-corrected two-sample *t*-tests yielded no significant group differences in the model parameters. At an uncorrected level, there was evidence for a higher learning rate ε for participants with obesity (*p*_uncorr_ = 0.036). Consistent with the analysis of learning performance, the Pavlovian parameter π did not significantly differ between groups.

**Table 2:** Parameter estimates from the computational reinforcement learning model.

**Parameter**	**Mean MAP normal-weight**	**Mean MAP obese**	***p*-value (uncorrected/** **Bonferroni corrected)**	**Effect size *d***
Learning rate ε	0.235	0.292	**0.036**/0.217	0.564
Irreducible noise ξ	0.063	0.062	0.680/1	0.109
Pavlovian parameter π	0.280	0.225	0.475/1	0.189
Bias *b*	0.545	0.442	0.597/1	0.140
Effectiveness of reward ρ_*R*_	7.143	7.193	0.875/1	0.042
Effectiveness of punishment ρ_*P*_	6.196	6.316	0.805/1	0.065

*Mean MAP estimates for parameters from the reinforcement learning model, p-values and effect sizes for the statistical comparison between participants with and without obesity. Results significant at p < 0.05 (two-tailed) are printed in bold letters*.

Supplementary Figure 1 shows a visualization of the individual parameter estimates. Additionally, the observed and simulated learning time courses are depicted in Supplementary Figure 2.

## Discussion

In the current study, we investigated the influence of reward and punishment predicting cues on instrumental approach and avoidance learning in individuals with obesity. We employed a paradigm in which participants learned to actively respond to a target stimulus or withhold an action, in order to gain an anticipated monetary reward or avoid monetary losses. We hypothesized that individuals with obesity would show a stronger corruption of instrumental learning by Pavlovian cues.

Expectedly, our results indicate that participants were generally better at learning to approach a reward or to withhold an active response when confronted with imminent punishment. However, comparing performance of participants with obesity and normal-weight participants contradicted our initial hypothesis. We found no conclusive evidence for a stronger bias on instrumental learning by cues of monetary gains or losses in individuals with obesity. Instead, obese participants showed better learning performance as indexed by higher learning rates and a higher number of correct actions. This was present for both approach and avoidance learning in the main task and also in an additional instrumental learning task, where Pavlovian influences were minimized.

To our knowledge, this study is the first to assess the corruption of instrumental *learning* by Pavlovian cues in individuals with obesity. However, some previous work examined influences on the *execution* of goal-directed behavior. Two studies found that individuals with obesity may be less sensitive to changes in reward value and thereby show stronger habitual than goal-directed responding (Horstmann et al., 2015b; Janssen et al., 2017). This must be contrasted with classical Pavlovian-instrumental-transfer tasks, which directly assess how cues associated with a reward alter existing instrumental behavior. In two studies employing these tasks, obese and normal-weight individuals exhibited similar response biases, while goal-directed behavior seemed to be more strongly affected by rewarding food cues in overweight participants (Lehner et al., 2017a; Meemken and Horstmann, 2019). Together, these results suggest that the presence of reward or punishment predicting cues *per se* does not bias the execution or learning of goal-directed responses more strongly in obese than normal-weight individuals.

Individuals with obesity showed *enhanced* instrumental learning with a slightly, but significantly higher number of correct responses (on average 3.3 more correct responses in 60 trials per condition). This was present across action and valence conditions and accompanied by an increased model-derived learning rate in individuals with obesity. We previously also found evidence for an increased reversal learning performance in obese individuals under specific task-conditions (Meemken et al., 2018). However, our current results stand in contrast with several other previous studies, which found an impaired performance when learning to predict food (Zhang et al., 2014) and non-food reinforcement (Coppin et al., 2014; Kastner et al., 2017; Mathar et al., 2017; Kube et al., 2018). Interestingly, in these studies obese individuals were not primarily impaired in learning the meaning of reward predicting cues. Rather, alterations were primarily found for the stimuli less or non-predictive of positive outcomes. For instance, Zhang et al. (2014) reported reduced differential conditioning when learning to predict food vs. no reward. The reduction was driven by increased reward expectancies toward cues that were in fact never paired with a reward. Outside of the food context, we (Mathar et al., 2017; Kube et al., 2018) and others (Coppin et al., 2014) have found that individuals with obesity may be slower or less successful in learning the meaning of unfavorable choice options and consequently learning to avoid them. This may be linked to impaired learning for outcomes that were worse than expected (negative prediction errors) and a reduced utilization of neural prediction error signals in individuals with obesity (Mathar et al., 2017). Obesity-related alterations in striatal dopamine D2 receptor signaling (Wang et al., 2001; Klein et al., 2007; Sevgi et al., 2015) or dopaminergic tone (Horstmann et al., 2015a) have been suggested to contribute to this.

As mentioned above, our observation of a better instrumental learning performance in obese individuals stands in contrast with the majority of previous studies (Coppin et al., 2014; Kastner et al., 2017; Mathar et al., 2017; Kube et al., 2018). Participant-specific characteristics that could explain these differential results (e.g., age, education, BMI), seem to be relatively consistent across studies and thus are not likely to have driven these differences. However, previous obesity studies have mainly used paradigms in which instrumental learning was defined as learning to choose stimuli based on their associations with a reward or punishment avoidance (e.g., Coppin et al., 2014; Kastner et al., 2017; Kube et al., 2018). While these tasks heavily rely on learning the value of the stimuli (termed stimulus-value learning), our task focused on the learning of action values (termed action-value learning). More specifically, our participants learned to choose between two actions to obtain a beneficial outcome. Both processes involve instrumental actions that subserve the maximization of rewards, but they are in fact distinct forms of learning with markedly different neural substrates. For instance, learning to choose between two stimuli has been shown to depend on the ventral striatum and orbitofrontal cortex, while choosing between different actions recruits the dorsal anterior cingulate cortex (Wunderlich et al., 2010; Camille et al., 2011; Rothenhoefer et al., 2017). Previous studies and our paradigm therefore likely addressed different aspects of instrumental learning. Individuals with obesity may be impaired in stimulus-value-based learning scenarios but show intact learning in action-based learning scenarios. Future studies should therefore include both types of instrumental learning paradigms. This way, one could directly test whether learning differences between normal-weight and obese individuals are indeed task-specific.

In addition to instrumental learning tasks, classical conditioning has received considerable attention in obesity research, as repeated overeating is thought to result in increased food cue responsiveness through conditioning processes (Berridge et al., 2010; Stice and Burger, 2018). Further, it has been suggested that individuals with obesity show a faster acquisition of appetitive responses toward food reward cues, i.e., they need fewer couplings of a cue and a reward to establish a conditioned response toward the predictive cue (van den Akker et al., 2017). Evidence on alterations in such Pavlovian learning processes is, however, mixed. Results range from intact (Meyer et al., 2015) to impaired conditioning (van den Akker et al., 2017) to a generalization of reward expectations to non-rewarded cues (Zhang et al., 2014) and learned preferences for cues not predictive of food reward (Coppin et al., 2014). Here, we tried to obtain a related measure for Pavlovian learning from monetary reward stimuli, but found no significant group differences between normal-weight and obese participants. The task was, however, comparatively brief and easy and may not have been sensitive enough to detect small group differences in Pavlovian learning performance.

A number of aspects should be considered in future studies. The model we used here has previously been shown to best fit participants' behavior in this task (Guitart-Masip et al., 2014). Thus, we adopt it in its current form to maintain comparability across studies. However, it primarily captures the impact of reward (monetary gain) and punishment (monetary losses) on behavioral adaptation, but disregards reward omission and punishment avoidance as alternative forms of feedback. Further, other applications of this paradigm have recently shown that the addition of an instrumental learning bias may further improve model fit (Swart et al., 2017). While the current Pavlovian bias is conceptualized as a response bias, the addition of a learning bias may e.g., capture a tendency to believe that a reward was more likely caused by action than inaction. Lastly, some previous studies found that overweight and moderately obese may be more distinct from normal-weight individuals in reward sensitivity than those with severe obesity (Davis et al., 2004; Dietrich et al., 2014). Relatedly, an inverted u-shaped relation between BMI and learning-related dopamine transmission has been suggested (Horstmann et al., 2015b). Future studies should therefore consider participants across the whole BMI range (including overweight).

The current study expands on the hypothesis that obesity is characterized by enhanced automatic action tendencies that can override goal-directed behavior in the light of anticipated rewards or punishments. In sum, we found no conclusive evidence for a stronger bias in individuals with obesity. Instead, contrary to previous studies, individuals with obesity showed a better instrumental learning performance. We therefore argue that individuals with obesity are not impaired in reinforcement learning *per se*. Instead, specific task characteristics may account for the differential results: Individuals with obesity may be impaired in tasks incorporating stimulus-value learning, but perform better in action-value learning scenarios. How this difference affects effective behavioral change and could potentially be used for behavioral interventions still has to be determined.

## Data Availability Statement

The datasets generated for this study are available on request to the corresponding author.

## Ethics Statement

The study was carried out in accordance with the Declaration of Helsinki and was approved by the ethics committee of the University of Leipzig.

## Author Contributions

JK, AV, and JN conceived of the study and designed it. SH and JK performed the measurements. JK, KW, and JN analyzed the data. JK, KW, SH, AV, and JN wrote the manuscript.

### Conflict of Interest

The authors declare that the research was conducted in the absence of any commercial or financial relationships that could be construed as a potential conflict of interest.
