# Supplementary material for: Enhanced Go and NoGo Learning in Individuals With Obesity
Source: Front Behav Neurosci. 2020 Feb 14;14:15. doi: 10.3389/fnbeh.2020.00015 (PMC7033453; doi:10.3389/fnbeh.2020.00015)
Supplement: Supplementary file 1 [file Table_1.docx]

Supplementary Material

# Personality, working memory and learning performance

Previous studies have identified obesity-related personality characteristics and potential working memory deficits that may mediate alterations in reinforcement-based learning processes (Aberg, Doell, & Schwartz, 2016; Collins & Frank, 2012; Coppin, Nolan-Poupart, Jones-Gotman, & Small, 2014; Dietrich, Federbusch, Grellmann, Villringer, & Horstmann, 2014; Kim, Yoon, Kim, & Hamann, 2014; Simon et al., 2010; Zhang, Manson, Schiller, & Levy, 2014). Thus, we aimed to investigate if group differences in PIL learning performance were mediated by differences in working memory capacity, impulsivity, reward and punishment sensitivity, or susceptibility of eating behaviour to environmental cues.

We found evidence for significant group differences between normal-weight and obese participants in punishment sensitivity (BIS/BAS-BIS) and susceptibility of eating behaviour to environmental cues (TFEQ disinhibition), but none of the other personality or working memory variables (Table 1). We thus added the main effects of BIS/BAS-BIS and TFEQ disinhibition into separate GEE models that additionally included all main and interaction effects of the predictors valence (reward, punishment), action (go, nogo), group (normal-weight, obese), and sex (male, female). We found no evidence for a mediating effect of BIS/BAS-BIS [main effect of BIS/BAS-BIS: Wald Χ^2^ = 0.165, p = .685] and TFEQ disinhibition [main effect of TFEQ-Disinhibition: Wald Χ^2^ = 0.374, p = .541], suggesting that obesity-related alterations in learning performance were unrelated to group differences in personality.

Further, we tested if personality and working memory characteristics that did not differ between groups, exhibited a general influence on learning performance. We similarly added the main effects of BIS-15, BIS/BAS-BAS and working memory scores into separate GEE models that additionally included all main and interaction effects of the predictors valence (reward, punishment), action (go, nogo), group (normal-weight, obese), and sex (male, female). Again, there was no evidence for a significant effect of personality or working memory capacity on learning performance [main effect of BIS-15 total score: Wald Χ^2^ = 0.629, p = .428; main effect of BIS/BAS-BAS: Wald Χ^2^ = 2.716, p = .099; main effect of digit span working memory score forward+backward: Wald Χ^2^ = 0.613, p = .434].

# Supplementary Figure 1

**Figure S1. Single subject MAPs for all reinforcement learning model parameters.** Single subject MAPs were derived from the reinforcement learning model. Each circle represents the parameter estimate of one individual.

# Supplementary Figure 2

**Figure S2.** Cumulative means of observed and simulated behavior across trials in obese and normal-weight participants. Parameters for the simulation were derived from the reinforcement learning model.

# References

Aberg, K. C., Doell, K. C., & Schwartz, S. (2016). Linking Individual Learning Styles to Approach-Avoidance Motivational Traits and Computational Aspects of Reinforcement Learning. *﻿PLoS ONE, 11,* e0166675. https://doi.org/10.1371/journal.pone.0166675

Collins, A. G. E., & Frank, M. J. (2012). How much of reinforcement learning is working memory, not reinforcement learning? A behavioral, computational, and neurogenetic analysis. *European Journal of Neuroscience*, *35*(7), 1024–1035. https://doi.org/10.1111/j.1460-9568.2011.07980.x

Coppin, G., Nolan-Poupart, S., Jones-Gotman, M., & Small, D. M. (2014). Working memory and reward association learning impairments in obesity. *Neuropsychologia*, *65*, 146–155. https://doi.org/10.1016/j.neuropsychologia.2014.10.004

Dietrich, A., Federbusch, M., Grellmann, C., Villringer, A., & Horstmann, A. (2014). Body weight status, eating behavior, sensitivity to reward/punishment, and gender: relationships and interdependencies. *Frontiers in Psychology*, *5*, 1073. https://doi.org/10.3389/fpsyg.2014.01073

Kim, S. H., Yoon, H. S., Kim, H., & Hamann, S. (2014). Individual differences in sensitivity to reward and punishment and neural activity during reward and avoidance learning. *Social Cognitive and Affective Neuroscience*, *10*(9), 1219–1227. https://doi.org/10.1093/scan/nsv007

Simon, J. J., Walther, S., Fiebach, C. J., Friederich, H., Stippich, C., Weisbrod, M., & Kaiser, S. (2010). Neural reward processing is modulated by approach- and avoidance-related personality traits. *NeuroImage*, *49*(2), 1868–1874. https://doi.org/10.1016/j.neuroimage.2009.09.016

Zhang, Z., Manson, K. F., Schiller, D., & Levy, I. (2014). Impaired associative learning with food rewards in obese women. *Current Biology*, *24*, 1731–1736. https://doi.org/10.1016/j.cub.2014.05.075
